# The Impact of Microbial Diversity on Biogenic Amines Formation in Grasshopper Sub Shrimp Paste During the Fermentation

**DOI:** 10.3389/fmicb.2020.00782

**Published:** 2020-04-24

**Authors:** Xue Sang, Kexin Li, Yaolei Zhu, Xinxiu Ma, Hongshun Hao, Jingran Bi, Gongliang Zhang, Hongman Hou

**Affiliations:** ^1^School of Food Science and Technology, Dalian Polytechnic University, Dalian, China; ^2^Liaoning Key Lab for Aquatic Processing Quality and Safety, Dalian Polytechnic University, Dalian, China

**Keywords:** grasshopper sub shrimp paste, fermentation, microbial diversity, biogenic amines, correlation analysis

## Abstract

Biogenic amines (BAs) and microbial diversity are important factors affecting food quality and safety in fermented foods. In this study, the bacterial and fungal diversity in grasshopper sub shrimp paste taken at different fermentation times were comprehensively analyzed, while the pH, colony counts, salinity, total volatile base nitrogen (TVB-N) and BA contents were quantitatively determined. In addition, the correlations among the samples with respect to microbial communities and the different parameters investigated especially BAs were also established. By combining the results of spearman correlation heatmap with the contents of BAs produced by the 102 halotolerant bacteria isolated from the grasshopper sub shrimp paste, six major genera of bacteria (*Jeotgalibaca*, *Jeotgalicoccus*, *Lysinibacillus*, *Sporosarcina*, *Staphylococcus*, and *Psychrobacter*) were found to be positively correlated with BA production level, suggesting that these bacteria might have a strong tendency to produce BAs. Other bacteria such as *Lentibacillus*, *Pseudomonas*, and *Salinicoccus* were considered as poor BA producers. The grasshopper sub shrimp paste was characterized by a relatively high abundance of *Tetragenococcus*, which was the dominant genus during the fermentation process, and it also produced a relatively high level of BAs but the spearman correlation heatmap revealed a negative correlation between *T. muriaticus* and BA level. Analysis of the species relevance network in grasshopper sub shrimp explained that the actual production of BAs by a certain strain was closely related to other species present in the complex fermentation system.

## Introduction

**FIGURE 1:**
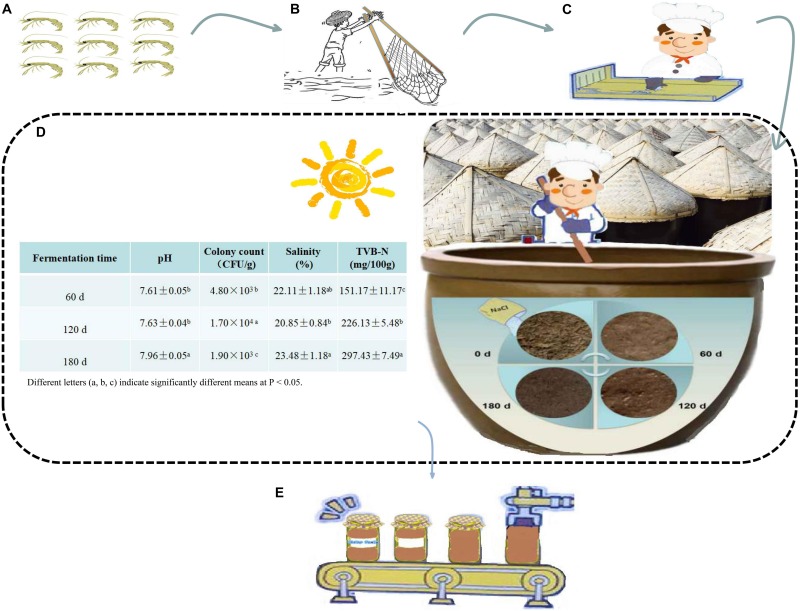
The fermentation process of grasshopper sub shrimp paste. **(A)** Grasshopper sub shrimp, **(B)** Catching grasshopper sub shrimp, **(C)** Screening process to remove the impurities, **(D)** Natural fermentation and changes in physicochemical and microbial indexes during fermentation, **(E)** Bottling.

Panjin shrimp paste is a famous traditional fermented aquatic product in China, especially pastes that are fermented from grasshopper sub shrimps. Grasshopper sub shrimps are found in the water that borders seawater and freshwater, the whole bodies are transparent and the longest length are only 0.008–0.01 m. Due to the tiny sizes of grasshopper sub shrimp, shrimp pastes that are made from them tend to have lower fat and cholesterol content, and higher astaxanthin and calcium levels than that made from other shrimps. Panjin is located in the southwestern part of Liaoning Province, the center of the Liaohe River Delta in China. It has the largest reed marsh in Asia and is the site of the world’s spectacular red beach. The geographical location of Panjin is very suitable for the growth of grasshopper sub shrimp. The shrimps are caught in April each year. The captured shrimps are first subjected to a screening process to remove the impurities. After that, put the shrimps into earthenware jars mixed with 15%–20% salt and stir them evenly. The jars with straw lids are left outdoor to allow fermentation to occur naturally over a period of about 180 days. Open the lids at least twice a day and stir them up and down with wooden sticks for 20 min to make them ferment evenly (Figure 1). The products are usually stored in glass or plastic bottles, sealed tightly and kept in cool places, with a shelf life of about 1 year. Grasshopper sub shrimp paste has high nutritional value, but it is susceptible to microbial contamination when it is processed in an open environment during production (Pongsetkul et al., 2014; Cai et al., 2017; Gu et al., 2018; Hao and Sun, 2020). Therefore, it is difficult to control the quality of the grasshopper sub shrimp paste. In addition to monitoring the basic parameters of the fermentation such as pH, salinity, colony count and TVB-N, the content of BAs is an indispensable criterion for determining whether the fermented food is safe for human consumption (Li et al., 2018; Park et al., 2019). Biogenic amines (BAs) are organic, basic, and nitrogenous compounds of low molecular weights that are present in plants, microbes, and animal cells, and they can be detected in raw as well as in fermented foods (Tittarelli et al., 2019). At high concentrations, BAs can cause some deleterious effects, especially histamine and tyramine, which can induce adverse effects like nausea, headaches and neurological disorders. Biogenic polyamines such as putrescine and cadaverine can enhance the toxicity of BA via interference with the detoxification mechanisms (Jeon et al., 2018). In view of the importance of BAs to human health and food safety, it is very important to monitor their contents in food. At present, high-performance liquid chromatography is the most suitable method for the analysis of fermented food (EFSA, 2011).

In recent years, there are some studies related to the impact of microbial diversity on BAs formation in fermented food, such as fish sauce, sausages, and soy sauce (Li et al., 2018, 2019; Wang et al., 2018). While shrimp paste from different manufacturers at home and abroad have been used as raw materials to study their biochemical, nutritional, sensory characteristics, and to findings from such studies have been used to screen for starter cultures (Cai et al., 2017; Pongsetkul et al., 2017; Kim et al., 2019), there has been no research investigating the production of BAs in grasshopper sub shrimp paste, and the connection between BA production and the quality of the paste. Grasshopper sub shrimp paste is rich in amino acids, providing a perfect environment for the production of BAs. Previously, it has been shown that the production and accumulation of BAs in fermented foods may be the result of a complex process and that it can be influenced by many factors and their interactions (Schirone et al., 2013; Gu et al., 2018; Hao and Sun, 2020). The relationship between BAs accumulation and microbial diversity at the different fermentation steps of grasshopper sub shrimp paste is still not well understood.

In this study, we investigated and identified the bacterial and fungi diversities in grasshopper sub shrimp paste produced in Panjin using an Illumina Miseq sequencing and cultured-dependent approaches. In addition, the correlation between the changes in BAs contents and the bacterial community at different stages of the fermentation process was also analyzed. Due to the formation of BAs is highly dependent on bacterial strains, therefore, we evaluated the ability of producing BAs of halophilic bacteria isolated from grasshopper sub shrimp paste samples, so as to more accurately reflect the relationship between strains and BAs-producing. Finally, the species correlation was examined at the genus level using species correlation network maps to reflect the interaction of species in the shrimp paste and the implication of such correlation was discussed.

## Experimental Methods

### Collection of Samples

Grasshopper sub shrimps in this study were collected from the junction of Bohai Sea and Liaohe River. The shrimp paste was prepared in a Panjin shrimp paste-processing plant using a local and natural fermentation method (Figure 1). After the screening process, put the shrimps into fermenters (earthenware jars, height 0.57 m, caliber 0.50 m, volume 75 kg) mixed with about 17% salt and stir them evenly. The fermenters with straw lids were left outdoor, and the fermentation was allowed to proceed for 180 days, the range of temperature during natural fermentation is about 10–50°C. Samples of the paste were collected at 60, 120, and 180 days after the initiation of fermentation. In order to ensure the uniformity and representativeness of the paste, the samples were collected from the upper, middle, and lower parts of 10 different fermenters (0.2 kg samples were taken from each part of each fermenter a time), and combined for subsequent analysis. All samples were aseptically collected into sterile disposable boxes, which were then placed in a container filled with ice packs and transported to the laboratory. A portion of the sample taken at each time point was immediately used for viable cell count and physicochemical and microbial assays, while the rest was kept at −80°C for DNA extraction and chemical analysis.

### Physicochemical and Microbial Measurements

The shrimp paste (10 g) was homogenized in 90 mL of distilled water in a stomacher (Stomacher 400 Circulator, Seward, Worthing, United Kingdom). The pH of the homogenate was recorded with a pH meter (FiveEasy Plus^TM^ FE28, Mettler Toledo, Shanghai, China). The salinity was measured according to Phewpan et al., 2020. The shrimp paste was also subjected to aerobic plate count performed according to the Microbiological Examination of National Food Safety Standard (Chinese Standard GB4789.2, 2016). TVB-N content in the shrimp paste was measured according to Chinese Standard GB5009.228 (2016).

### Analysis of Microbial Diversity by Culture-Independent Technique

Microbial DNA was extracted from the shrimp paste using the E.Z.N.A.^®^ soil DNA Kit (Omega Bio-Tek, Norcross, GA, United States). Bacteria and fungi in the paste were detected by PCR using primers specific for bacterial 16S rRNA and fungal ITS rRNA genes, respectively. The V3-V4 region of bacterial 16S rRNA genes was amplified using the primers 338F (5′-ACTCCTACGGGAGGCAGCAG-3′) and 806R (5′′-GGACTACHVGGGTWTCTAAT-3′). The fungal internal transcribed spacer one (ITS1) hypervariable region of fungal ITS rRNA genes was amplified using the primers ITS1(5′-CTTGGTCATTTAGAGGAAGTAA-3′) and ITS2(5′-GCTGCGTTCTTCATCGATGC-3′). The resulting PCR products were extracted from a 2% agarose gel and further purified using the AxyPrep DNA Gel Extraction Kit (Axygen Biosciences, Union City, CA, United States) and quantified with a QuantiFluor^TM^-ST Kit (Promega, Madison, WI, United States) according to the manufacturer’s protocol.

Purified amplicons were pooled in equimolar and paired-end sequenced (2 × 300) on an Illumina MiSeq platform (Illumina, San Diego, CA, United States) according to the standard protocols by Majorbio Bio-Pharm Technology Co., Ltd. (Shanghai, China).

The raw data of bacteria and fungi had been uploaded to the NCBI website, with the accession number of SRP222988 and SRP222991, respectively.

### Analysis of Bacterial Diversity by Culture-Dependent Method

#### Isolation and Selection of Halotolerant Bacteria From Grasshopper Sub Shrimp Paste

The shrimp paste (10 g) was homogenized in 90 mL of distilled water inside a sterile stomacher bag and pummeled for 1 min using a high-speed beating homogenizer (Stomacher^®^ 400 Circulator, Seward, Worthing, United Kingdom). After that, the homogenate was serially diluted 10^–1^–10^–6^ fold with distilled water and 200 μL of each dilution was plated on Luria-Bertani (LB), de Man Rogosa, and Sharp (MRS) agar plates, each supplemented with 14% and 10% (w/v) NaCl. The LB and MRS agar plates were incubated at 37°C for 48 h. Colonies were chosen for further analysis, and isolates were enriched by subculturing in the corresponding agar plates for at least four times. The pure strain was stored at −80°C in the corresponding liquid culture medium containing 32% (v/v) glycerin.

#### Genotypic Identification

The genomic DNA was extracted using a TIANamp bacteria DNA Kit (TIANGEN, Beijing, China), performed according to the manufacturer’s protocol.

To identify the bacteria, the sequence of 16S rRNA of each pure isolate was sequenced. The universal primer pair, 27F and 1492R (Weisburg et al., 1991) was used to amplify the 16S rRNA genes under the following PCR conditions: initial denaturation at 94°C for 4 min, followed by 32 cycles of 94°C for 30 s, 58°C for 30 s and 72°C for 45 s, and a final elongation step at 72°C for 5 min.

The PCR products were sequenced by BGI (Shenzhen, China). The identification of closely related species and the pairwise nucleotide similarity of the 16S rRNA gene sequences were calculated using EzBioCloud server (Yoon et al., 2017).

#### BAs Determination by HPLC

The identity and concentration of the BAs in the grasshopper sub shrimp paste were determined based on a previously described procedure (Hu et al., 2012). An aliquot (20 mL) of 10% trichloroacetic acid (TCA) was added to 5 g of shrimp paste, and the mixture was homogenized using a vortex mixer, and then allowed to react at 4°C for 2 h. After that, the mixture was centrifuged at 3000 × *g* and 4°C for 10 min. The supernatant was collected, whereas the residue was extracted again with an equal volume of 10% TCA and centrifuged as before. The two supernatants were combined, and the final volume was adjusted to 50 mL with 10% TCA, and then filtered through a 0.22 μm-pore-size filter.

Bacteria isolated from the sub shrimp paste were first cultured and the BA produced was determined according to a previously described method (Jeon et al., 2018). A loopful of the bacteria was inoculated into 5 mL of LB broth containing 0.5% L -histidine monohydrochloride monohydrate, L -Tyrosine disodium salt hydrate, L -ornithine monohydrochloride, and L -lysine monohydrochloride (pH 5.8). The culture was supplemented with 0.0005% pyridoxal-HCl (Sangon Biotech, Shanghai, China). After incubation at 37°C for 24 h, 100 μL of the culture was transferred to a new test tube containing 5 mL of the same medium and then incubated at 37°C for another 24 h followed by two more subculturings. Next, 100 μL of the culture was transferred to a new test tube containing 5 mL of the same medium and incubated at 37°C for 48 h. A 9-mL aliquot of 10% TCA was then added to 1 mL of the culture inside a 50-mL centrifuge tube. The sample was mixed by vortexing and then allowed to react at 4°C for 2 h followed by centrifugation at 3000 × *g* at 4°C for 10 min. The supernatant was filtered through a 0.22 μm-pore-size filter.

Derivatization of BAs was carried out according to the procedure developed by Ben-Giglrey et al. (1998). One milliliter of the extract or standard solution prepared above was mixed with 200 μL of 2 M sodium hydroxide and 300 μL of saturated sodium bicarbonate. This was followed by the addition of 2 mL of dansyl chloride solution (10 mg/mL) prepared in acetone and the mixture was incubated at 45°C for 40 min. The residual dansyl chloride was removed by adding 125 μL of ammonia followed by further incubation at room temperature for 30 min. After that, the volume was adjusted with acetonitrile to a final volume of 5 mL. Finally, the mixture was centrifuged at 3000 × *g* for 5 min, and the supernatant was filtered twice through a 0.22-μm filter. The filtered supernatant was kept at −80°C for subsequent HPLC analysis.

The quantification of BA was carried out using an Agilent 1260 HPLC unit (Agilent technologies Inc., Santa Clara, CA, United States) which consisted of an Agilent Zorbax SB-C18 column (4.6 × 150 mm) coupled to a quaternary pump and a diode array detector. A 20-μL sample was injected into the column at a flow rate of 1.0 mL/min. The column was eluted with a binary solution consisting of ultrapure water (solvent A) and acetonitrile (solvent B) using the following optimized gradient: 0–10 min, 55% B; 10–15 min, 55–65% B; 15–20 min, 65–80% B; 20–25 min, 80% B; 25–30 min, 80–90% B; 30–33 min, 90% B; 33–35 min, 90–55% B. The column temperature was set as 30°C and the eluent was monitored by absorbance at 254 nm. Quantitative determination of BA was carried out using an external standard method of peak area. The content of BA was expressed as mg/kg or mg/L. All experiments were in triplicate.

### Statistical Analysis

All statistical analyses were performed using the IBM SPSS Statistics 22 program, and Origin 2018 software. Some data were analyzed using the free online platform of Major Bio i-Sanger Cloud Platform^1^.

## Results

### Changes in Physicochemical Indexes During the Fermentation of Grasshopper Sub Shrimp Paste

The results of pH, colony count, salinity and TVB-N for the 60 days-, 120 days-, 180 days- samples are shown in Figure 1. The fermentation was roughly divided into three stages: Stage I – 0–60 days; Stage II – 60–120 days, and Stage III – 120–180 days. During fermentation, the shrimp paste became darker and more fluid. The pH (the pH of 60 days, 120 days, 180 days samples were 7.61 ± 0.05, 7.63 ± 0.04, and 7.96 ± 0.05, respectively), and the salinity (the salinity of 60 days, 120 days, 180 days samples were 22.11 ± 1.18, 20.85 ± 0.84, and 23.48 ± 1.18%, respectively), both initially fluctuated and then increased with increased fermentation time, whereas colony counts (the colony counts of 60 days, 120 days, 180 days samples were 4.80 × 10^3^, 1.70 × 10^4^, and 1.90 × 10^3^ CFU/g, respectively), increased and then decreased. As for TVB-N, it exhibited an increasing trend (varied from 151.17 ± 11.17 to 297.43 ± 7.49 mg/100 g).

### Dynamics of the Bacterial Community

**FIGURE 2:**
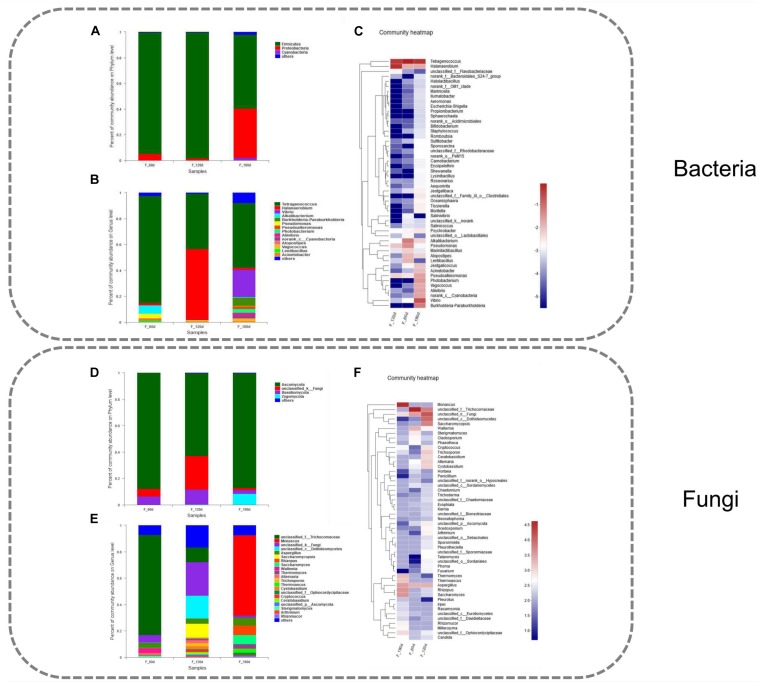
Community composition of bacteria and fungi in grasshopper sub shrimp paste samples during fermentation. **(A)** Relative abundance of bacterial phyla, **(B)** Relative abundance of bacterial genus, **(C)** Heatmap dendrogram showing the diversity of bacteria at genus level, **(D)** Relative abundance of fungal phyla, **(E)** Relative abundance of fungal genus, **(F)** Heatmap dendrogram showing the diversity of fungi at genus level.

Bacterial 16S rRNA gene sequences were classified according to the phylum and genus levels in order to investigate the composition of the bacterial community in the grasshopper sub shrimp paste. A total of 21 phyla and 302 genera were detected. The relative abundance of each of the three particular phyla, Firmicutes, Proteobacteria, and Cyanobacteria, was higher than 1% (Figure 2A). Among the 302 genera, the relative abundance of each of the 14 genera exceeded 1%. The top 50 species, which accounted for a total abundance of about 98.88% at the genus level, were selected for community analysis, and the result revealed different types and quantities of bacteria at the different stages of fermentation (Figure 2C). *Tetragenococcus* appeared to be a stable dominant genus throughout the entire fermentation, and therefore, it constituted the key bacterial group in grasshopper sub shrimp paste. Its abundance decreased from 83% in the 60-days samples to 42.7% in the 120-days samples but increased to 50% in the 180-days samples (Figure 2B). To our best knowledge, this is the first time that bacteria belonging to the genus *Tetragenococcus* have been found to account for such a high percentage in a fermented product. The change in *Halanaerobium* abundance was in contrast to that of *Tetragenococcus*. The abundance of *Halanaerobium* increased from 1.52% in the 60-days samples to 54.86% in the 120-days samples but decreased to 1.67% in the 180-days samples. In addition to *Tetragenococcus* and *Halanaerobium*, *Alkalibacterium*, *Pseudomonas*, *Vibrio*, *Burkholderia-Paraburkholderia*, *Pseudoalteromonas*, *Photobacterium*, *Aliivibrio* and *norank_c_Cyanobacteria* were also among the top 10 genera (Figure 2B).

### Dynamics of the Fungal Community

The fungal ITS gene sequences were classified at both the phylum and genus levels in order to investigate the composition of the fungal community in grasshopper sub shrimp paste. A total of 7 phyla and 202 genera were identified in the grasshopper sub shrimp paste. Four of the phyla exhibited more than 1% relative abundance, and these phyla were Ascomycata, Unclassified_k_Fungi, Basidiomycota, and Zygomycota (Figure 2D). Among the 202 genera detected, 38 exhibited more than 1% relative abundance. Thus, a large number of *unclassified_k_Fungi* and *Aspergillus* were detected in the 60-, 120-, and 180-days samples (Figure 2F), but the abundance of *unclassified_k_Fungi* initially increased and then decreased, while the abundance of *Aspergillus* remained relatively unchanged over the entire course of fermentation.

*Monascus*, *Saccharomyces*, *Thermomyces*, *Thermoascus*, *Wallemia*, *Rhizomucor*, *Millerozyma*, *Irpex*, and *Rasamsonia* all exhibited a significant increase in abundance in the180-days samples compared with the 60-days, and 120-days samples. The abundance of *Monascus* increased significantly in the 180-days samples compared with the 60-days and 120-days samples, while that of *unclassified_f_Trichocomaceae* decreased significantly (Figure 2E).

### Analysis of Bacterial Diversity by Culture-Dependent Method

A total of 102 strains of bacteria were identified from the shrimp paste and they were classified into 31 genera and 50 species based on their 16S rRNA sequences (Table 1). The five most abundant genera were *Bacillus* (16 isolates), *Tetragenococcus* (14 isolates), *Jeotgalicoccus* (11 isolates), *Lentibacillus* (10 isolates), and *Staphylococcus* (6 isolates).

**TABLE 1:** Identification of bacteria based on 16S rRNA sequencing.

Genera	Species	Numbers
*Agrobacterium*	*Agrobacterium sp.*	1
*Alkalibacillus*	*Alkalibacillus salilacus*	2
*Allobacillus*	*Allobacillus halotolerans*	1
*Acinetobacter*	*Acinetobacter lwoffii*	1
*Bacillus*	*Bacillus aerius*	1
	*Bacillus aquimaris*	1
	*Bacillus aryabhattai*	1
	*Bacillus cereus*	2
	*Bacillus encimensis*	1
	*Bacillus horikoshii*	1
	*Bacillus megaterium*	2
	*Bacillus pacificus*	1
	*Bacillus pumilus*	3
	*Bacillus thuringiensis*	1
	*Bacillus wiedmannii*	1
	*Bacillus zhangzhouensis*	1
*Bhargavaea*	*Bhargavaea indica*	5
*Cellulosimicrobium*	*Cellulosimicrobium marinum*	1
	*Cellulosimicrobium sp.*	1
*Carnobacterium*	*Carnobacterium sp.*	1
*Dermacoccus*	*Dermacoccus barathri*	1
	*Dermacoccus sp.*	1
*Gracilibacillus*	*Gracilibacillus halotolerans*	1
*Jeotgalibaca*	*Jeotgalibaca dankookensis*	1
*Jeotgalicoccus*	*Jeotgalicoccus halotolerans*	9
	*Jeotgalicoccus nanhaiensis*	2
*Klebsiella*	*Klebsiella variicola*	1
*Kocuria*	*Kocuria carniphila*	3
*Lentibacillus*	*Lentibacillus juripiscarius*	9
	*Lentibacillus salicampi*	1
*Lysinibacillus*	*Lysinibacillus macroides*	1
*Microbacterium*	*Microbacterium maritypicum*	1
*Micrococcus*	*Micrococcus sp.*	1
*Oceanobacillus*	*Oceanobacillus picturae*	1
*Paenibacillus*	*Paenibacillus ihumii*	1
	*Paenibacillus lautus*	3
	*CP009282_s*	1
*Planococcus*	*Planococcus citreus*	1
*Pisciglobus*	*Pisciglobus halotolerans*	1
*Psychrobacter*	*MRYB_s*	3
*Pseudomonas*	*Pseudomonas stutzeri*	1
*Rhizobium*	*Rhizobium pusense*	1
*Salinicoccus*	*Salinicoccus salsiraiae*	2
	*Salinicoccus sp.*	2
*Sporosarcina*	*Sporosarcina saromensis*	1
*Staphylococcus*	*Staphylococcus epidermidis*	5
	*Staphylococcus haemolyticus*	1
*Tetragenococcus*	*Tetragenococcus muriaticus*	14
*unclassified Bacillaceae*	*Bacillaceae bacterium*	1
*Virgibacillus*	*Virgibacillus halodenitrificans*	1
	*Virgibacillus jeotgali*	1

### Biogenic Amine Content of Grasshopper Sub Shrimp Paste

Eight common BAs [tryptamine (Try), β-phenethylamine (Phe), putrescine (Put), cadaverine (Cad), histamine (His), tyramine (Tyr), spermine (Spm), and spermidine (Spd)] were detected and the types and contents of BA varied with different fermentation times (Table 2). Put and Tyr were detected only in the 180-days samples. Try was detected in the 120-days samples, and its content in the 180-days samples increased significantly. Similarly, the contents of Put and Cad also increased significantly in the 180-days samples. The contents of Cad and His increasedduring the fermentation, but the content of His was low and increased only slightly.

**TABLE 2:** Changes in BA contents in grasshopper sub shrimp paste samples during fermentation.

	Biogenic amines (mg/L)		
	60 days	120 days	180 days
Try	ND	20.96 ± 0.12^b^	77.20 ± 1.90^a^
Phe	ND	ND	ND
Put	ND	ND	61.92 ± 1.17
Cad	0.29 ± 0.13^c^	3.05 ± 0.18^b^	73.58 ± 1.08^a^
His	2.41 ± 0.23^b^	2.63 ± 0.27^b^	3.65 ± 0.32^a^
Tyr	ND	ND	4.60 ± 0.10^a^
Spm	ND	ND	ND
Spd	ND	ND	ND

*Data are presented as mean ± SDs of three replicates; ND – not detected. (a, b, c) Different letters in the same row indicate a significant difference (P ≤ 0.05).*

### Production of Biogenic Amines by Halotolerant Bacteria

**FIGURE 3:**
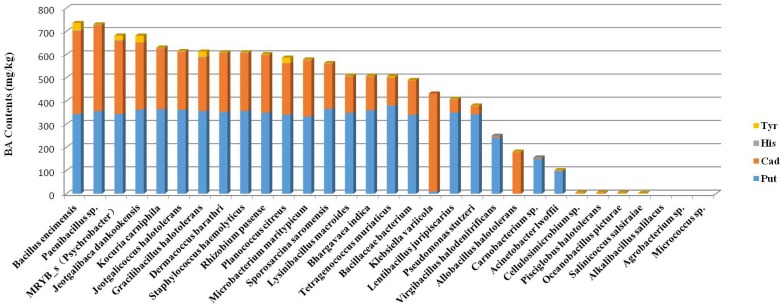
Ranking of the total amount of BAs produced by different species.

BAs produced by the 102 strains were detected by HPLC. For each species examined, the strains that produced the highest amount of total BAs were sequenced. Different strains were found to produce different types of BA and at different concentrations, but all strains appeared to produce Put and Cad as the main BAs (Figure 3).

### RDA/CCA Analysis of Samples, Microbial Communities, Physicochemical and Microbial Indexes

**FIGURE 4:**
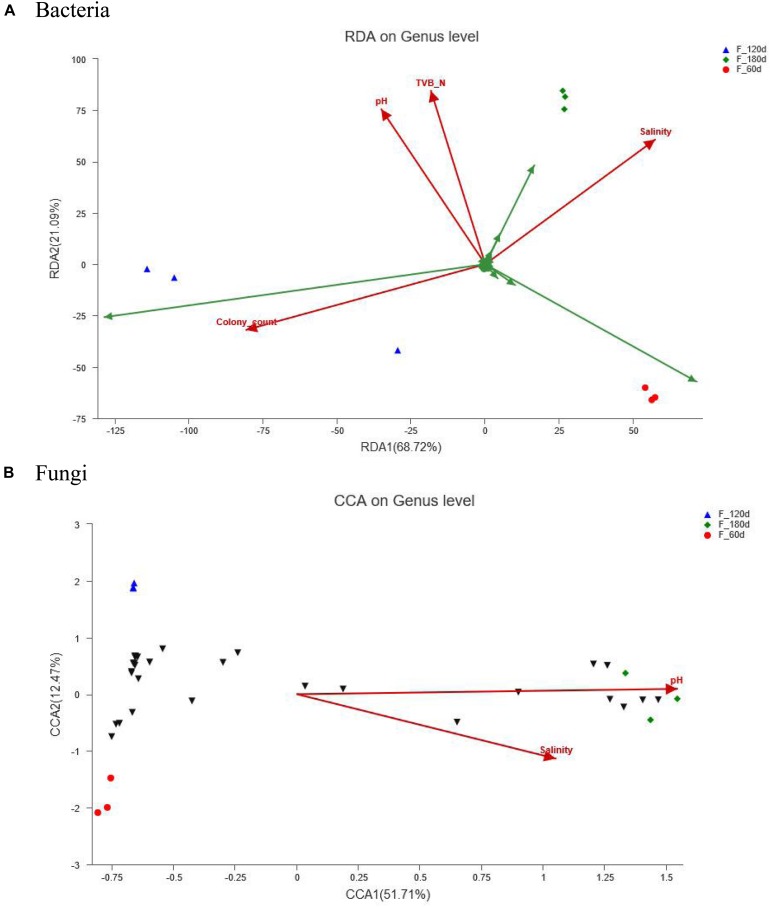
RDA/CCA analysis of samples, microbial communities and physicochemical indexes: **(A)** Bacteria, **(B)** Fungi. Red arrows represent quantitative environmental factors, the length of which can represent the degree of impact of environmental factors on the distribution of species with the microbial communities; the angle represents correlation (acute angle: positive correlation, the smaller the angle, the greater the correlation; obtuse angle: negative correlation, the bigger the angle, the greater the correlation; right angle: no correlation).

RDA/CCA is a sort method of linear/single peak model, based on the result obtained from a combined correspondence and multiple regression analyses, which can analyze the relationship among samples, microbial communities, physicochemical and microbial indexes. The effects exerted by pH, TVB-N and salinity on the distribution of bacterial and fungal communities in the shrimp paste tended to vary, depending on the duration of the fermentation. When the paste was fermented for 180 days, the effects exerted by pH, TVB-N and salinity were equally important in determining the distribution of the bacterial community, while the colony count had the greatest impact in the 120-days samples (Figure 4A). The colony count data was positively correlated with pH and TVB-N, but negatively correlated with salinity. And there was a significant positive correlation between pH and TVB-N. As for the fungal community, only the effects exerted by the pH and salinity were important to the distribution of fungal community when the paste was fermented for 180 days (Figure 4B).

### Analysis of the Correlation Between Bacterial Community and Biogenic Amines

**FIGURE 5:**
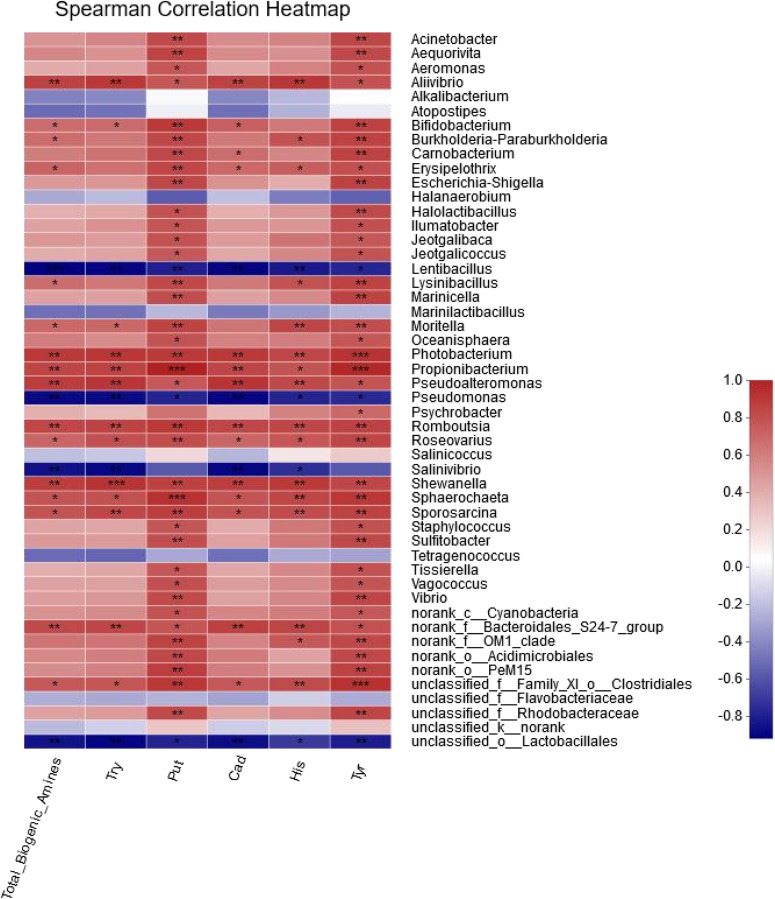
The correlation between bacterial community and biogenic amines in different fermentation times.

The correlation between BAs and the 50 most abundant genera was evaluated by the spearman correlation heatmap. Almost every genus exhibited a certain degree of correlation with BAs (Figure 5). By comparing the genera that were positively correlated with BA in the spearman correlation heatmap with the 31 genera identified from the shrimp paste Table 1, eight common genera were found to be positively correlated with BAs (Supplementary Table 1), and they were *Acinetobacter* (0.536 < *R* < 0.906), *Carnobacterium* (0.523 < *R* < 0.915), *Jeotgalibaca* (0.630 < *R* < 0.788), *Jeotgalicoccus* (0.5 < *R* < 0.788), *Lysinibacillus* (0.692 < *R* < 0.852), *Sporosarcina* (0.704 < *R* < 0.941), *Staphylococcus* (0.593 < *R* < 0.915), and *Psychrobacter* (0.408 < *R* < 0.650). Four common genera were negatively correlated with BAs, and they were *Lentibacillus* (−0.775 < *R* <−0.955), *Pseudomonas* (−0.893 < *R* <−0.749), *Tetragenococcus* (−0.704 < *R* <−0.453), and *Salinicoccus* (−0.481 < *R* ≤ 0).

According to the concentrations of BA produced by the halotolerant bacteria, *Jeotgalibaca dankookensis*, *Jeotgalicoccus halotolerans*, *Lysinibacillus macroides*, *Sporosarcina saromensis*, *Staphylococcus haemolyticus*, and *MRYB_s(Psychrobacter*)appeared to have strong BA-producing ability (Figure 3). It is worth noting that *Tetragenococcus* was the absolute dominant genus found in grasshopper sub shrimp paste. *Tetragenococcus muriaticus* was the only species belonging to genus *Tetragenococcus* that was isolated from grasshopper sub shrimp paste in this study, and the result conformed to the species diagram (Supplementary Figure 1). *T. muriaticus* produced a relatively high level of BAs, but the spearman correlation heatmap revealed a negative correlation between *T. muriaticus* and BA level. Therefore, it is necessary to build a species correlation network by calculating the correlation between species to explain this phenomenon.

### Analysis of Species Relevance Network Map

**FIGURE 6:**
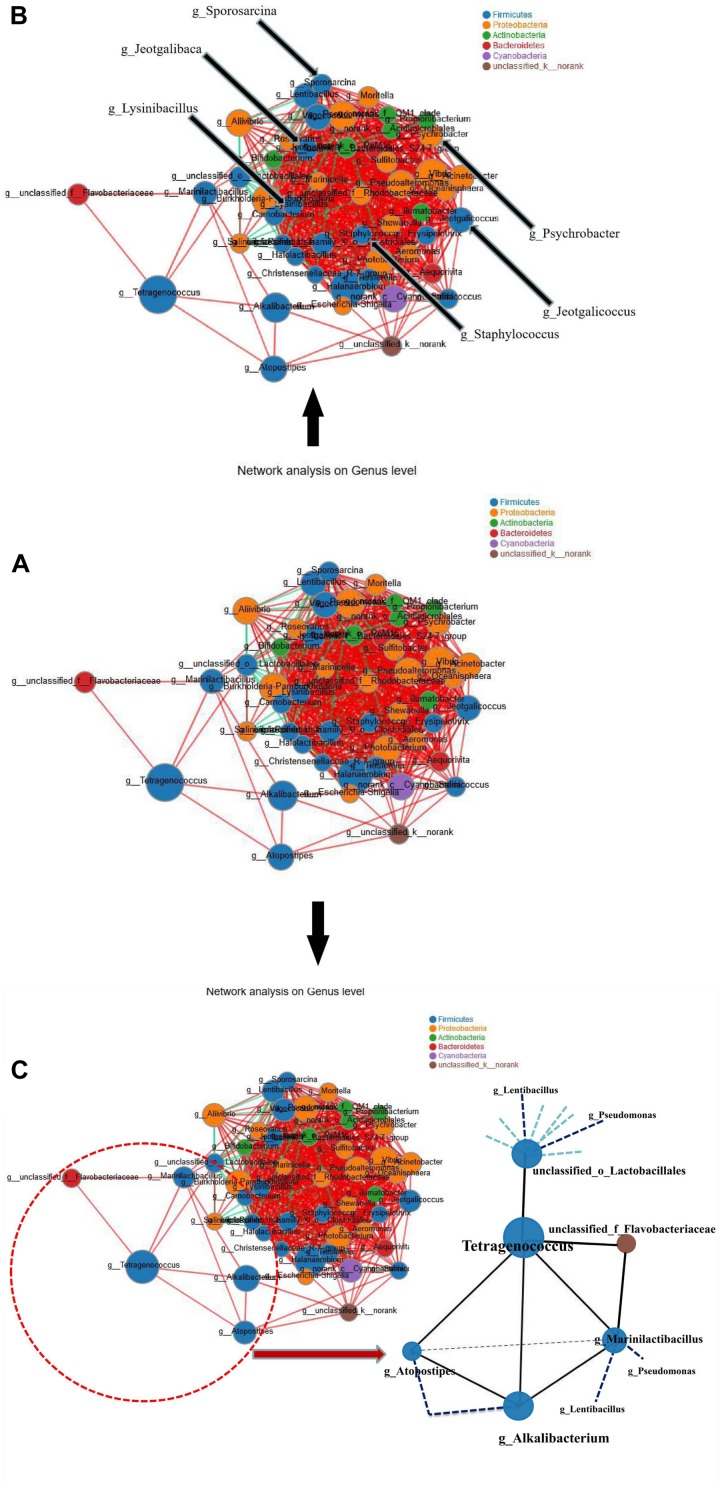
Species relevance network map. **(A)** The interaction among the species in the shrimp paste samples. **(B)** The location of *Jeotgalibaca*, *Jeotgalicoccus*, *Lysinibacillus*, *Sporosarcina*, *Staphylococcus* and *Psychrobacter*. **(C)** The interaction between *Tetragenococcus* and its correlated genera. (The figure shows the species with *p* < 0.05, the absolute value of correlation coefficient ≥0.5; The size of nodes indicates the abundance of species, and different colors indicate different species. The red lines indicate positive correlation, while the green lines indicate negative correlation. The thicker the line, the higher the correlation between species, and the more lines, the closer the relationship between the species).

Species relevance network maps in this study mainly reflect species correlation and interaction at different taxonomic levels under the natural fermentation conditions. We selected the 50 most abundant genera and calculated the spearman rank correlation coefficient to reflect the correlation among the species. The interaction among the species in the shrimp paste samples was shown in Figure 6A. In the species relevance network map, degree represents the number of nodes connected with a node, clustering indicates the connection between a node and its neighboring nodes. If a node is completely connected with its neighboring nodes, the clustering coefficient is 1. On the contrary, if a node has almost no connection with its neighboring nodes, the clustering coefficient is close to 0. The larger the clustering coefficient is, the more important of the node. The six genera which were found to be positively correlated with BA production level, *Jeotgalibaca*, *Jeotgalicoccus*, *Lysinibacillus*, *Sporosarcina*, *Staphylococcus*, and *Psychrobacter* (indicated by arrows in Figure 6B) were located at the center of the network graph, the degrees were 32, 35, 36, 29, 31, and 27, respectively. The clustering coefficients were 0.89, 0.79, 0.75, 0.75, 0.88, 0.89, respectively. For *Tetragenococcus*, the degree and clustering coefficient of were 5 and 0.40, respectively (Supplementary Table 2). The six genera (*Jeotgalibaca*, *Jeotgalicoccus*, *Lysinibacillus*, *Sporosarcina*, *Staphylococcus*, and *Psychrobacter*) played important roles in the fermentation system, while *Tetragenococcus* was relatively weak.

*Tetragenococcus* was found to be positively correlated with five genera, *Alkalibacterium*, *Atopostipe*s, *unclassified_f_Flavobacteriaceae*, *unclassified_o_Lactobacillales* and *Marinilactibacillus* (Figure 6C). Taking the node of *Tetragenococcus*, the location of *Tetragenococcus* could be found at the edge, and connected with the whole bacterial network through *Lactobacillales* and unclassified_k__norank (another connection node between *Alkalibacterium* and *Atopostipes).* According to the results of the spearman correlation heatmap (Figure 5), the five genera, especially *Lactobacillales*, were all negatively correlated with BAs.

## Discussion

The production of grasshopper sub shrimp paste is a complex process influenced by multifarious factors. During the fermentation process, the change of pH is a comprehensive reflection of the metabolism of microorganisms. The production of lactic acid and acetic acid by different species of lactic acid bacteria (LAB) (Steinkraus, 2010) results in the decrease of pH, while the formation of metabolites or volatile compounds (Pongsetkul et al., 2016) results in the increase of pH. In this study, the pH fluctuation in the early stages of fermentation may be due to the production of varies metabolites. As a result of the increase of LAB abundance in Stage III, a significantly increase in pH was observed, which might be mainly caused by the significant increase of basic nitrogenous substances such as ammonia and amines, and it was consistent with the results of RDA analysis that pH was significantly positive correlated with TVB-N (Figure 4A). Grasshopper sub shrimp paste is a Chinese traditional fermented salted food, its high salinity is not only conducive to the storage of products, but also the most important factor limiting the selection and evolution of microbiota (Mokashe et al., 2018). In the early stages of fermentation, the colony counts showed an upward trend, which was due to the relatively rich nutrients, which were conducive to the growth of bacteria. In the later stage of fermentation, hot weather coupled with daily hand stirring can cause a large loss of water, resulting in an increase in salinity. With the significant increase of salinity in Stage III, salt intolerant bacteria were inhibited, and the total number of colonies was significantly reduced, it was also consistent with the results of RDA analysis. According to Chinese Aquatic Industry Standard SC/T3602 (2016) and Chinese Domestic Trade Industry Standard SB/T10525 (2009) prescribed for salted shrimp paste, the salinity, TVB-N and the colony counts should be less than or equal to 25%, 450 mg/100 g and 8 × 10^3^CFU/g, respectively. The results in this study showed that all the indicators determined in the grasshopper sub shrimp paste samples were up to standard.

Based on 16S rRNA gene sequence, *Tetragenococcus* appeared to be a stable dominant genus, *Tetragenococcus* consists of LAB which can tolerate the high concentration of salt in the fermented foods and syrups, such as fish sauce (Chuea-nongthon et al., 2017), cheese (Haastrup et al., 2018), jeotgal (Song et al., 2018), and soybean products (Chen et al., 2018; Li et al., 2018). In many fermented products, *Tetragenococcus* plays a crucial role in the production of nutrients and flavors (Jeyaram et al., 2008; Chen et al., 2012). During the fermentation, LAB produced CO_2_, which gave rise to an anaerobic environment, allowing for an increase in the relative abundance of some anaerobic and intestinal bacteria at a later stage of the fermentation (120-days samples). The change in *Halanaerobium* abundance was in contrast to that of *Tetragenococcus*. The dynamic changes of the two genera corresponded to the above changes in pH, salinity, and colony count. During fermentation, the number of species increased significantly, with *Vibrio*, *Burkholderia-Parburkholderia*, *Pseudoalteromonas*, *Photobacterium*, *Aliivibrio*, *norank_c_Cyanobacteria*, *Vagococcus*, *Acinetobacter*, *Jeotgalicoccus* exhibited a marked increase in the 180-days samples. The abundance of *Alkalibacterium*, *Pseudomonas*, *Marinilactibacillus*, and *Lentibacillus* decreased during fermentation. These strains are also commonly found in other fermented food (Namwong et al., 2005; Pakdeeto et al., 2007; Jeon et al., 2018; Santiyanont et al., 2019).

Fungi play a positive role in the production of nutrient, flavor and texture of grasshopper sub shrimp paste. For examples, molds produce different proteases, peptidase, amylase, and precursors of aromatic substances (Wang et al., 2005; Yan et al., 2013) while yeasts can transform glucose to glycerol, ethanol, isobutyl alcohol, isoamyl alcohol, and other substances, contributing to the formation of flavor (Gao et al., 2013; Yan et al., 2013; Song et al., 2015). *Aspergillus* plays a major role in the process of sauce production because of its significant presence in meju, soybean, douchi, and doenjang (Yoo et al., 1999; Choi et al., 2003; Li et al., 2018; Jeong et al., 2019). *Monascus* is a genus of mold, and among the known species of this genus, the red-pigmented *Monascus purpureus* is very important because of its use in the production of certain fermented foods in East Asia, particularly in China and Japan. *Trichocomaceae* is a family of fungi belonging to the order Eurotiales, which can adapt to extreme environmental conditions. Members of this family are globally distributed, being ubiquitous in the soil, and a common partner of decaying plants and food. This family contains some of the most common fungi such as *Penicillium* and *Aspergillus* (Li et al., 2014).

The culture-dependent method identified 31 genera from the shrimp paste, 12 genera were among the 50 most abundant genera obtained from high-throughput sequencing. The most abundant genera isolated by culture-dependent method were *Bacillus.* However, *Bacillus* was not among the 50 most abundant genera obtained from high-throughput sequencing, although *Bacillus* is considered to be key microbes in several soybeans-based fermentations and *Bacillus* also confers various positive effects on human health. For instance, some species of *Bacillus* are able to produce various fungicidal agents, amylases, hyaluronic acid, and plasmin, which not only play an important role in fermentation, but also confer a health effect on humans (Zhao et al., 2009; He et al., 2013). Several studies have suggested that the growth of *Bacillus cereus* is inhibited by the presence of LAB because of the acidic environment created as a result of the increased growth of LAB (Cai et al., 2014). Bacilli did not constitute a major microbiota in grasshopper sub shrimp paste, but their effect on the flavor and nutrition of grasshopper sub shrimp paste and the interaction between *Bacilli* and LAB require further study (Gu et al., 2018). The difference between the two methods may be due to the culture condition being unsuitable for the growth of some bacteria, greatly reduced viability of some bacterial cells in the samples, or that the cells were already dead at the beginning of the culturing period. This indicated that the cultured-dependent method could easily lead to the selective growth of some bacteria, masking the true status of the microbial community structure (Santiyanont et al., 2019).

There are many kinds of BA and these have different degrees of toxicity in human, and the toxic effect appears to vary from person to person, because of the differences in sensitivity and tolerance toward BA as well as the fact that the metabolic pathways of different BAs are different (Varela et al., 2014). In addition, the toxicity of BA is also affected by many external factors, such as the presence of other BAs and amine oxidases. Although there are no regulations governing the permissible BA content in most foods, the United States FDA has recommended an upper limit of 50 mg/kg for histamine in the edible portion of fish and the EFSA panel considers fish containing histamine at less than 50 mg/kg to be safe for consumption (EFSA, 2011). The maximum limit of histamine in fish such as mackerel permitted by law is 200–400 mg/kg in China, and 200 mg/kg in South Korea. Previous studies have shown that BAs tend to vary in their toxicity, and suggested that an upper limit for the following BAs: 30 mg/kg for β-phenylethylamine; 100 mg/kg for histamine, 100–800 mg/kg for tyramine (Brink et al., 1990); and a total BA content in food not exceeding 900 mg/kg (Brink et al., 1990; Yang et al., 2014), since a total BA content exceeding 1000 mg/kg may cause serious harm to human health (Xia et al., 2016). In comparison, the contents of the different BAs detected in grasshopper sub shrimp paste all appeared to be within the safe range.

The correlation between BA and the 50 most abundant genera was evaluated by the spearman correlation heatmap. Almost every genus exhibited a certain degree of correlation with BA. By comparing the genera that were positively correlated with BA in the spearman correlation heatmap with the 31 genera identified from the shrimp paste, eight common genera were found to be positively correlated with BAs, and four common genera were negatively correlated with BAs. According to the concentrations of BA produced by the 102 halotolerant bacteria, *Jeotgalibaca dankookensis*, *J. halotolerans*, *L. macroides*, *S. saromensis*, *S. haemolyticus*, and MRYB_s(*Psychrobacter*)appeared to have strong BA-producing ability. It is worth noting that *T. muriaticus* was the only species belonging to genus *Tetragenococcus* and also produced a relatively high level of BA, but the spearman correlation heatmap revealed a negative correlation between *T. muriaticus* and BA level. Li et al., 2019 had found a similar situation, it is necessary to analyze the species network to explain this phenomenon.

In the species relevance network map, *Tetragenococcus* was found at the edge of the network and to be positively correlated with five genera, *Alkalibacterium*, *Atopostipe*s, *unclassified_f_Flavobacteriaceae*, *unclassified_o_Lactobacillales*, and *Marinilactibacillus*, according to the results of the spearman correlation heatmap, the five genera, especially *unclassified_o_Lactobacillales*, were negatively correlated with BAs. Therefore, in the complex fermentation system, the production of the actual BAs by a certain strain might not only be affected by different environments, but could also be closely related to other species. Even if these species could produce BA, they might not have been able to play a role in a complex fermentation system. Members of the genus *Tetragenococcus* are moderately halophilic homofermentative LAB, which can tolerate high salinity (in the presence of 18% NaCl or greater) and are frequently found in salted fermented foods such as fish sauce and soy sauce (Jeon et al., 2018; Wang et al., 2018). These bacteria might play important roles in the synthesis of amino acid and in improving the flavor of the product during fermentation (Kim et al., 2019).

In summary, the bacterial and fungal diversities, BAs contents in grasshopper sub shrimp paste at different fermentation times were mainly comprehensive analyzed, and possible correlations were also discussed. Due to provide theoretical data, we evaluated the ability of producing BAs of halophilic bacteria isolated from grasshopper sub shrimp paste, and explained the actual production of BA by a particular strain was closely related to other species present in the complex fermentation system through the species relevance network analysis. To our knowledge, this has been the first study that explored a large microbial diversity during the fermentation process of grasshopper sub shrimp paste of Chinese origin. It provided important insights into the microbiota and BAs content of grasshopper sub shrimp paste.

## Data Availability Statement

The raw data of bacteria and fungi had been uploaded to the NCBI website, with the accession number of SRP222988 and SRP222991, respectively.

## Author Contributions

HHo and XS designed this study. XS conducted the experiments. KL, YZ, and XM performed the data analyses, HHo contributed reagents and materials. XS, HHa, JB, and GZ drafted and revised the manuscript. All authors read and approved the final version of this manuscript.

## Conflict of Interest

The authors declare that the research was conducted in the absence of any commercial or financial relationships that could be construed as a potential conflict of interest.

## Footnotes

1 www.i-sanger.com

## References

[B1] Ben-Giglrey B. Vieites B. Juan M. Villa T. G. Barros-Velazquez J. (1998). Changes in biogenic amines and microbiological analysis in albacore (*Thunnus alalunga*) muscle during frozen storage. *J. Food Prot.* 61 608–615. 10.4315/0362-028x-61.5.608 9709235

[B2] Brink B. Ten Damink C. Joosten H. M. L. J. Huis Veld J. H. (1990). Occurrence and formation of biologically active amines in foods. *Int. J. Food Microbiol.* 11 73–84. 10.1016/0168-1605(90)90040-C 2223522

[B3] Cai L. Wang Q. Dong Z. Liu S. Zhang C. Li J. (2017). Biochemical, nutritional, and sensory quality of the low salt fermented shrimp paste. *J. Aquat. Food Prod. T.* 26 706–718. 10.1080/10498850.2016.1276111

[B4] Cai X. Ma J. Wei D.-Z. Lin J.-P. Wei W. (2014). Functional expression of a novel alkaline-adapted lipase of *Bacillus amyloliquefaciens* from stinky tofu brine and development of immobilized enzyme for biodiesel production. *Anton. Leeuw. Int. J.* 106 1049–1060. 10.1007/s10482-014-0274-5 25199563

[B5] Chen T. Jiang S. Xiong S. Wang M. Zhu D. Wei H. (2012). Application of denaturing gradient gel electrophoresis to microbial diversity analysis in Chinese Douchi. *J. Sci. Food Agric.* 92 2171–2176. 10.1002/jsfa.5604 22318896

[B6] Chen Y.-H. Liu X.-W. Huang J.-L. Baloch S. Xu X. Pei X.-F. (2018). Microbial diversity and chemical analysis of shuidouchi, traditional Chinese fermented soybean. *Food Res Int.* 116 1289–1297. 10.1016/j.foodres.2018.10.018 30716918

[B7] Chinese Aquatic Industry Standard SC/T3602 (2016). *Salted Shrimp Paste (in Chinese).* Beijing: China Standard Press of China.

[B8] Chinese Domestic Trade Industry Standard SB/T10525 (2009). *Shrimp Sauce (in Chinese).* Beijing: China Standard Press of China.

[B9] Chinese Standard GB4789.2 (2016). *Determination of Total Colonies In Food Microbiological Test. (in Chinese).* Beijing: China Standard Press of China.

[B10] Chinese Standard GB5009.228. (2016). *Determination of Volatile Salt Nitrogen In Food (in Chinese).* Beijing: China Standard Press of China.

[B11] Choi K.-K. Cui C.-B. Ham S.-S. Lee D.-S. (2003). Isolation, identification and growth characteristics of main strain related to meju fermentation. *J. Korean Soc. Food Sci. Nutr.* 32 818–824. 10.3746/jkfn.2003.32.6.818

[B12] Chuea-nongthon C. Rodtong S. Yongsawatdigul J. Steele J. L. (2017). Draft genome sequences of *Tetragenococcus muriaticus* strains 3MR10-3 and PMC-11-5 isolated from Thai fish sauce during natural fermentation. *Genome Announc.* 5:e00198-17 10.1128/genomeA.00198-17 PMC5391428 28408690

[B13] EFSA, (2011). Scientific opinion on risk based control of biogenic amine formation in fermented foods. *EFSA J.* 9:2393 10.2903/j.efsa.2011.2393

[B14] Gao X. Liu H. Yi X. Liu Y. Wang X. Xu W. (2013). Microbial floral dynamics of Chinese traditional soybean paste (Doujiang) and commercial soybean paste. *J. Microbiol. Biotechnol.* 23 1717–1725. 10.4014/jmb.1306.06004 24002452

[B15] Gu J. Liu T. Sadiq F. A. Yang H. Yuan L. Zhang G. (2018). Biogenic amines content and assessment of bacterial and fungal diversity in stinky tofu - A traditional fermented soy curd. *LWT Food Sci. Technol.* 88 26–34. 10.1016/j.lwt.2017.08.085

[B16] Haastrup M. K. Johansen P. Malskaer A. H. Castro-Mejia J. L. Kot W. Krych L. (2018). Cheese brines from danish dairies reveal a complex microbiota comprising several halotolerant bacteria and yeasts. *Int. J. Food Microbiol.* 285 173–187. 10.1016/j.ijfoodmicro.2018.08.015 30176565

[B17] Hao Y. Sun B. (2020). Analysis of bacterial diversity and biogenic amines content during fermentation of farmhouse sauce from Northeast China. *Food Control.* 108:106861 10.1016/j.foodcont.2019.106861

[B18] He S. Ma Y. Zhou S. Song W. Wang R. (2013). Antioxidant activities of fermented soybean prepared with Bacillus subtilis. *Asian J. Chem.* 25 10565–10568. 10.14233/ajchem.2013.15988

[B19] Hu Y. Huang Z. Li J. Yang H. (2012). Concentrations of biogenic amines in fish, squid and octopus and their changes during storage. *Food Chem.* 135 2604–2611. 10.1016/j.foodchem.2012.06.121 22980848

[B20] Jeon A. R. Lee J. H. Mah J.-H. (2018). Biogenic amine formation and bacterial contribution in Cheonggukjang, a Korean traditional fermented soybean food. *LWT Food Sci. Technol.* 92 282–289. 10.1016/j.lwt.2018.02.047

[B21] Jeong D. W. Lee H. Jeong K. Kim C. T. Shim S. T. Lee J. H. (2019). Effects of starter candidates and NaCl on the production of volatile compounds during soybean fermentation. *J. Microbiol. Biotechnol.* 29 191–199. 10.4014/jmb.1811.11012 30602270

[B22] Jeyaram K. Mohendro Singh W. Premarani T. Devi A. R. Chanu K. S. Talukdar N. C. (2008). Molecular identification of dominant microflora associated with ‘Hawaijar’ - a traditional fermented soybean (*Glycine* max (L.)) food of Manipur, India. *Int. J. Food. Microbiol.* 122 259–268. 10.1016/j.ijfoodmicro.2007.12.026 18281117

[B23] Kim K. H. Lee S. H. Chun B. H. Jeong S. E. Jeon C. O. (2019). Tetragenococcus halophilus MJ4 as a starter culture for repressing biogenic amine (cadaverine) formation during saeu-jeot (salted shrimp) fermentation. *Food Microbiol.* 82 465–473. 10.1016/j.fm.2019.02.017 31027807

[B24] Li F. Wang B. Wang L. Cao B. (2014). Phylogenetic analyses on the diversity of *Aspergillus fumigatus* sensu lato based on five orthologous loci. *Mycopathologia* 178 163–176. 10.1007/s11046-014-9790-0 25106755

[B25] Li L. Ruan L. Ji A. Wen Z. Chen S. Wang L. (2018). Biogenic amines analysis and microbial contribution in traditional fermented food of Douchi. *Sci. Rep.* 8:12567. 10.1038/s41598-018-30456-z 30135497 PMC6105706

[B26] Li L. Zou D. Ruan L. Wen Z. Chen S. Xu L. (2019). Evaluation of the biogenic amines and microbial contribution in traditional Chinese sausages. *Front. Microbiol.* 10:872 10.3389/fmicb.2019.00872 PMC6510162 31130922

[B27] Mokashe N. Chaudhari B. Patil U. (2018). Operative utility of salt-stable proteases of halophilic and halotolerant bacteria in the biotechnology sector. *Int. J. Biol. Macromol.* 117 493–522. 10.1016/j.ijbiomac.2018.05.217 29857102

[B28] Namwong S. Tanasupawat S. Smitinont T. Visessanguan W. Kudo T. Itoh T. (2005). Isolation of *Lentibacillus salicampi* strains and *Lentibacillus juripiscarius* sp. nov. from fish sauce in Thailand. *Int. J. Syst. Evol. Microbiol.* 55 315–320. 10.1099/ijs.0.63272-0 15653893

[B29] Pakdeeto A. Tanasupawat S. Thawai C. Moonmangmee S. Kudo T. Itoh T. (2007). *Lentibacillus kapialis* sp. nov., from fermented shrimp paste in Thailand. *Int. J. Syst. Evol. Microbiol.* 57:6. 10.1099/ijs.0.64315-0 17267980

[B30] Park Y. K. Lee J. H. Mah J. H. (2019). occurrence and reduction of biogenic amines in traditional asian fermented soybean foods: a review. *Food Chem.* 278 1–9. 10.1016/j.foodchem.2018.11.045 30583348

[B31] Phewpan A. Phuwaprisirisan P. Takahashi H. Ohshima C. Lopetcharat K. Techaruvichit P. (2020). Microbial diversity during processing of Thai traditional fermented shrimp paste, determined by next generation sequencing. *LWT Food Sci. Technol.* 122:108989 10.1016/j.lwt.2019.108989

[B32] Pongsetkul J. Benjakul S. Sampavapol P. Osako K. Faithong N. (2014). Chemical composition and physical properties of salted shrimp paste (*Kapi*) produced in Thailand. *Int. Aquat Res.* 6 155–166. 10.1007/s40071-014-0076-4

[B33] Pongsetkul J. Benjakul S. Sumpavapol P. Osako K. Faithong N. (2016). Properties of salted shrimp paste (Kapi) from acetes vulgaris as affected by postmortem storage prior to salting. *J. Food Process. Preserv.* 40 636–646. 10.1111/jfpp.12643

[B34] Pongsetkul J. Benjakul S. Vongkamjan K. Sumpavapol P. Osako K. (2017). Microbiological and chemical changes of shrimp acetes vulgaris during Kapi production. *J. Food Sci. Technol.* 54 3473–3482. 10.1007/s13197-017-2804-4 29051642 PMC5629156

[B35] Santiyanont P. Kanittha Chantarasakhaa L. Tepkasikul P. Srimarut Y. Mhuantong W. Tangphatsornruang S. (2019). Dynamics of biogenic amines and bacterial communities in a Thai fermented pork product Nham. *Food Res. Int.* 119 110–118. 10.1016/j.foodres.2019.01.060 30884638

[B36] Schirone M. Tofalo R. Fasoli G. Perpetuini G. Corsetti A. Manetta A. (2013). High content of biogenic amines in Pecorino cheeses. *Food Microbiol.* 34 137–144. 10.1016/j.fm.2012.11.022 23498190

[B37] Song E. J. Lee E. S. Park S. L. Choi H. J. Roh S. W. Nam Y. D. (2018). Bacterial community analysis in three types of the fermented seafood, jeotgal, produced in South Korea. *Biosci. Biotechnol. Biochem.* 82 1444–1454. 10.1080/09168451.2018.1469395 29742980

[B38] Song Y.-R. Jeong D.-Y. Baik S.-H. (2015). Monitoring of yeast communities and volatile flavor changes during traditional Korean soy sauce fermentation. *J. Food Sci.* 80 M2005–M2014. 10.1111/1750-3841.12995 26302401

[B39] Steinkraus K. H. (2010). Fermentations in world food processing. *Compr. Rev. Food Sci. Food Saf.* 1 23–32. 10.1111/j.1541-4337.2002.tb00004.x 33451246

[B40] Tittarelli F. Perpetuini G. Di Gianvito P. Tofalo R. (2019). Biogenic amines producing and degrading bacteria: a snapshot from raw ewes’ cheese. *LWT Food Sci. Technol.* 101 1–9. 10.1016/j.lwt.2018.11.030

[B41] Varela M. Alexiou G. Liakopoulou M. Papakonstantinou E. Pitsouni D. Alevizopoulos G. (2014). Monoamine metabolites in ventricular CSF of children with posterior fossa tumors: correlation with tumor histology and cognitive functioning. *J. Neurosurg. Pediatr.* 13 375–379. 10.3171/2014.1.PEDS13425 24559277

[B42] Wang R. Chau S. J. Law R. Webb C. (2005). Protease production and conidiation by Aspergillus oryzae in four fermentation. *Process. Biochem.* 40 217–227. 10.1016/j.procbio.2003.12.008

[B43] Wang Y. Li C. Li L. Yang X. Wu Y. Zhao Y. (2018). Effect of bacterial community and free amino acids on the content of biogenic amines during fermentation of Yu-lu, a Chinese fermented fish sauce. *J. Aquat. Food Prod. Trans.* 27 496–507. 10.1080/10498850.2018.1450573

[B44] Weisburg W. G. Barns S. M. Pelletier D. A. Lane D. J. (1991). 16S ribosomal DNA amplification for phylogenetic study. *J. Bacteriol.* 173 697–703. 10.1128/jb.173.2.697-703.1991 1987160 PMC207061

[B45] Xia X. Zhang Q. Zhang B. Zhang W. Wang W. (2016). Insights into the biogenic amine Metabolic landscape during industrial semi-dry Chinese rice wine fermentation. *J. Agric. Food Chem.* 64 7385–7393. 10.1021/acs.jafc.6b01523 27622644

[B46] Yan Y.-Z. Qian Y.-L. Ji F.-D. Chen J.-Y. Han B.-Z. (2013). Microbial composition during Chinese soy sauce koji-making based on culture dependent and independent methods. *Food Microbiol.* 34 189–195. 10.1016/j.fm.2012.12.009 23498197

[B47] Yang J. Ding X. Qin Y. Zeng Y. (2014). Safety assessment of the biogenic amines in fermented soya beans and fermented bean curd. *J. Agric. Food Chem.* 62 7947–7954. 10.1021/jf501772s 25029555

[B48] Yoo S. K. Cho W. H. Kang S. M. Lee S. H. (1999). Isolation and identification of microorganisms in Korean traditional soybena paste and soybean sauce. *J. Microbiol. Biotechn.* 27 113–117. 10.1016/j.jcrysgro.2011.12.009

[B49] Yoon S. H. Ha S. M. Kwon S. Lim J. Kim Y. Seo H. (2017). Introducing EzBioCloud: a taxonomically united database of 16S rRNA gene sequences and whole-genome assemblies. *Int. J. Syst. Evol. Microbiol.* 67 1613–1617. 10.1099/ijsem.0.001755 28005526 PMC5563544

[B50] Zhao J. Dai X. Liu X. Chen H. Tang J. Zhang H. (2009). Changes in microbial community during Chinese traditional soybean paste fermentation. *Int. J. Food Sci. Tech.* 44 2526–2530. 10.1111/j.1365-2621.2009.02079.x

