# Supplementary material for: The Impact of Microbial Diversity on Biogenic Amines Formation in Grasshopper Sub Shrimp Paste During the Fermentation
Source: Front Microbiol. 2020 Apr 24;11:782. doi: 10.3389/fmicb.2020.00782 (PMC7193991; doi:10.3389/fmicb.2020.00782)
Supplement: Supplementary file 1 [file Data_Sheet_1.doc]

Supplementary Material

**The impact of microbial diversity on biogenic amines formation in grasshopper sub shrimp paste during the fermentation**

Xue Sang, Kexin Li, Yaolei Zhu, Xinxiu Ma, Hongshun Hao, Jingran Bi, Gongliang Zhang, Hongman Hou

*** Correspondence:** Hongman Hou: houhongman@dlpu.edu.cn

# Supplementary Data


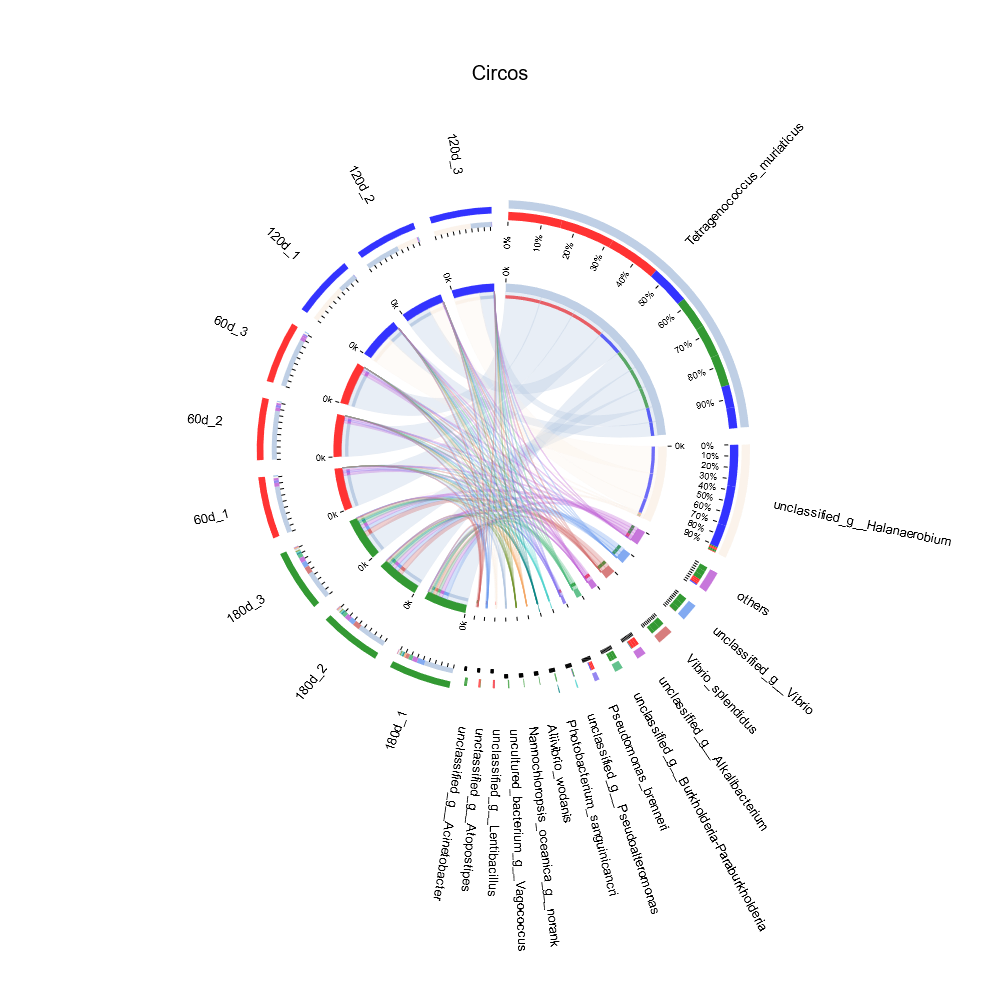


**Supplementary Figure 1.** Circos samples and species diagram on species level

**Supplementary Table 1.** Organism identified at the genus level in both the spearman correlation heatmap and the culture-dependent method.

| No. | **Genus** | Significance associated with BA |  |
| --- | --- | --- | --- |
| 1 | *Acinetobacter* | Put****,** Tyr** | Positive correlation |
| 2 | *Carnobacterium* | Put****，**Cad***，**Tyr** |
| 3 | *Jeotgalibaca* | Put***，**Tyr* |
| 4 | *Jeotgalicoccus* | Put***，**Tyr* |
| 5 | *Lysinibacillus* | Put****，**Cad***，**Tyr** |
| 6 | *Sporosarcina* | Put*****，**His***，**Tyr****，**Try* |
| 7 | *Staphylococcus* | Put***，**Tyr* |
| 8 | *Psychrobacter* | Tyr* |
|  |  |  |  |
| 9 | *Lentibacillus* | Try****，**Put****，**Cad****，**His****，**Tyr* | Negative correlation |
| 10 | *Pseudomonas* | Try****，**Put***，**Cad****，**His***，**Tyr* |
| 11 | *Tetragenococcus* | weak |
| 12 | *Salinicoccus* | weak |

Significance associated with BA: * 0.01 < *P* < 0.05, ** 0.001 < *P* < 0.01, *** *P* < 0.001.

**Supplementary Table 2.** The property sheet of network node.

| **node name** | **degree** | **clustering** |
| --- | --- | --- |
| *g__Jeotgalibaca* | 32 | 0.89 |
| *g__Jeotgalicoccus* | 35 | 0.79 |
| *g__Lysinibacillus* | 36 | 0.75 |
| *g__Psychrobacter* | 27 | 0.89 |
| *g__Sporosarcina* | 29 | 0.75 |
| *g__Staphylococcus* | 31 | 0.88 |
| *g__Tetragenococcus* | 5 | 0.40 |
